# The Predictive Levels of Serum Anti-Müllerian Hormone and the Combined Index of the Number of Retrieved Oocytes and Good-Quality Embryos in Advanced-Age Infertile Women

**DOI:** 10.1155/2022/4224417

**Published:** 2022-04-18

**Authors:** Tie-Cheng Sun, Xi Chen, Cheng Shi, Li Tian, Shan-Jie Zhou

**Affiliations:** ^1^Reproductive Medical Center, Department of Obstetrics and Gynecology, Peking University International Hospital, Beijing 102206, China; ^2^Reproductive Medical Center, Department of Obstetrics and Gynecology, Peking University People's Hospital, Beijing 100044, China

## Abstract

The primary objective of the study was to assess the values of serum anti-Müllerian hormone (AMH) levels and the combined index for the prediction of number of oocytes retrieved (NOR) and number of good-quality embryos (GQE) in infertile women undergoing IVF/ICSI treatment. A group of 521 infertile women aged 21–46 years were recruited as subject in this study. Serum AMH, hormones, and antral follicle count (AFC) were measured. The infertile women were categorized into three groups: 21–34 years (reproductive age), 35–39 years (reproductive age), and 40–46 years (advanced-age infertile). The predictive accuracy of variables was analyzed by the receiver operating characteristic (ROC) curve. AFC, AFC/age ratio, AMH/age ratio, and ovarian response prediction index (ORPI) decreased gradually, while AMH decreased significantly with increase in age. Moreover, NOR and GQE were positively correlated with AFC, AMH, AFC/age ratio, AMH/age ratio, and ORPI (*P* < 0.001). A statistical significance was observed in predicted oocyte retrieval including AMH, AMH/age ratio, and ORPI between 21–34 years and 35–46 years; especially in the 35–46 years group, these variables reached a “high” grade in the diagnostic accuracy because area under curve (AUC) ranged from 0.982 to 0.988 significantly. No statistical significance was observed for FSH, AMH, AFC, and related combined index predicting GQE. The predictive value of AFC and AFC/age ratio was limited regarding oocyte retrieval; however, AMH, AMH/age ratio, and ORPI concurrently had an excellent value for predicting NOR in reproductive-age women, especially in advanced-age infertile women.

## 1. Introduction

There is a strong association between NOR and the clinical miscarriage rate where the possibility of success is largely determined by ovarian response, the numbers of oocytes retrieved (NOR), and numbers of good-quality embryos (GQE) when infertile women carried out the cycles of in vitro fertilization and embryo transfer/intracytoplasmic sperm injection (IVF-ET/ICSI) [1]. The reliable markers of ovarian reserve provide an accurate estimation of NOR and GQE in the IVF/ICSI cycles of infertile women. The markers of diminished ovarian reserve and poor ovarian response, including female age, basal serum follicle-stimulating hormone (FSH), estradiol (E2), anti-Müllerian hormone (AMH), inhibin B, antral follicle counts (AFC), and ovarian volume, are used in ovarian stimulation during IVF/ICSI [2–4]. Usually, basal serum FSH level at day 3 was used to predict ovarian reserve, and level >10 IU/liter was considered consistent with poor ovarian response. However, compared with AMH, basal serum FSH was not independently associated with ovarian response, NOR, and GQE [4–7].

Recently, AMH has been used to assess ovarian reserve parameters and response to gonadotrophin stimulation, reproductive outcomes of infertile women [4, 7–10]. AMH is an emerging and one of the strongest markers of NOR during IVF cycles [9, 11, 12]. Recent studies indicated that AMH could also independently predict pregnancy outcomes [13–17]. Although a good predictive value both for AFC and AMH is reported, but published evidence leans towards AMH level. Due to its objectivity and potential standardization, as well as the convenience of testing at any time during the menstrual cycle, AMH is the gold standard biomarker for assessing ovarian reserve and predicting ovarian response [18].

In addition, the combined index of related AMH, such as AMH/age ratio and ovarian response prediction index (ORPI), indicated excellent effectiveness in predicting number of oocytes retrieved (NOR) and ovarian response [19, 20].

Although the relationship between AMH and NOR and good-quality embryos (GQE) in IVF treatment has been explored extensively [11, 12, 21–23], fewer trails are available regarding the predictive values of AMH and the combined index of related AMH for NOR and GQE, specially for the advanced-age infertile women. This study aimed to assess the values of the abovementioned parameters (AMH, AMH/age ratio, and ORPI) for women undergoing controlled ovarian stimulation for IVF/ICSI treatment.

## 2. Materials and Methods

### 2.1. Patients Selection and Study Design

This study retrospectively recruited 521 infertile women (aged 22–43 years) who were conceived via IVF/ICSI at Peking University People's Hospital from September 2015 to February 2017. The collected data included maternal age, reproductive hormonal profiles, AFC, NOR, and GQE, paternal age, and semen parameters. The inclusion criteria were age >18 years, no history of ovarian surgery, both ovaries present, no evidence of endocrine disorders, and no severe endometriosis. The exclusion criteria were medications within 12 weeks (for example, clomiphene, letrozole, and gonadotropins), patients with autoimmune diseases, cancer, and genetic diseases before ovarian stimulation protocols, and other endocrine diseases. Women were categorized into three groups based on their age: group 1 (21–34 years, *n* = 64), group 2 (35–39 years, *n* = 296), and group 3 (40–46 years, *n* = 161).

### 2.2. Ovarian Stimulation Protocols

All groups received ovarian stimulation using a standard luteal downregulation regimen (long protocol), flare-up short regimen (short protocol), and GnRH antagonist protocols [24, 25]. Oocyte retrieval was performed 36 h after self-administered subcutaneous injections of human chorionic gonadotropin (hCG), and embryo score was measured according to Istanbul consensus. The score was assessed, and the embryos were graded as of good morphology and were considered for GQE [26].

### 2.3. Measurement of Antral Follicle Counts (AFC)

The measurement of AFC was performed by experienced and qualified sonographers using the Philips HD11XE ultrasound system (Philips Ultrasound, Inc., Bothell WA, USA) to measure the diameter of the follicle on days 2–4 of menstrual cycle, and the total number of follicles (measuring 2–10 mm) on both ovaries was measured and defined as the total AFC.

### 2.4. Assessment of Reproductive Hormones and AMH

Serum samples were drawn on days 2–4 of a spontaneous natural cycle. The samples were separated within one hour of blood draw and stored at − 80 °C until analysis for follicle-stimulating hormone (FSH), luteinizing hormone (LH), E2, total testosterone (TT), and AMH.

The commercial kits and electrochemiluminescence assays available were used to estimate reproductive hormones levels (Abbott Ireland Diagnostics Division, Lisnamuck, Longford Co., Longford, Ireland) according to manufacturer's instructions. AMH from blood serum was measured using a commercially AMH detection kit (Elecsys® from Roche AMH assay, Roche Diagnostics, Mannheim, Germany).

In vitro fertilization (IVF) and intracytoplasmic sperm injection (ICSI), oocyte retrieval, fertilization, embryo culture, embryo scoring, blastocyst grade, and embryo transfer (ET) were carried out according to previously described in detail [20].

### 2.5. Calculation of Combined Index

All patients were measured to calculate body mass index (BMI) using a formula weight (kg)/height^2^ (m^2^). FSH/LH ratio, AFC/age ratio, AMH/age ratio, and ORPI were calculated using their levels, the number of antral follicles, and the age (years) of the patients. The ORPI was a simple three-variable index, and their equation is as follows: ORPI = (AMH x AFC)/patient age [19].

### 2.6. Semen Examination

Following the principles of the WHO laboratory manual [27], the semen samples should be collected after a minimum of two days and a maximum of seven days of sexual abstinence, and the semen parameters were calculated via computer-aided sperm analysis.

### 2.7. Statistical Analysis

Statistical analysis was done by using SPSS (version 18.0) for Windows (SPSS Inc., Chicago, USA) and MedCalc Statistical Software version 19.7.2 (MedCalc Software Ltd, Ostend, Belgium; http://www.medcalc.org). Statistical analyses were performed with one-way ANOVA. Pearson's correlation coefficients were used to assess the correlation throughout. Receiver operating characteristic (ROC) curves were constructed to examine the predictive accuracy of variables and the performance of ORPI in predicting clinical pregnancy. Measurements of the area under the curve (AUC), sensitivity, and specificity were used to evaluate the predictive models. ROC analysis was also calculated the area under the curve (AUC) and cutoff value. The differences between AUC of different parameters were compared using Fisherʼs *Z*-test. *P* value <0.05 was considered as statistically significant.

## 3. Results

### 3.1. Baseline Clinical and Biochemical Characteristics

**Figure 1:**
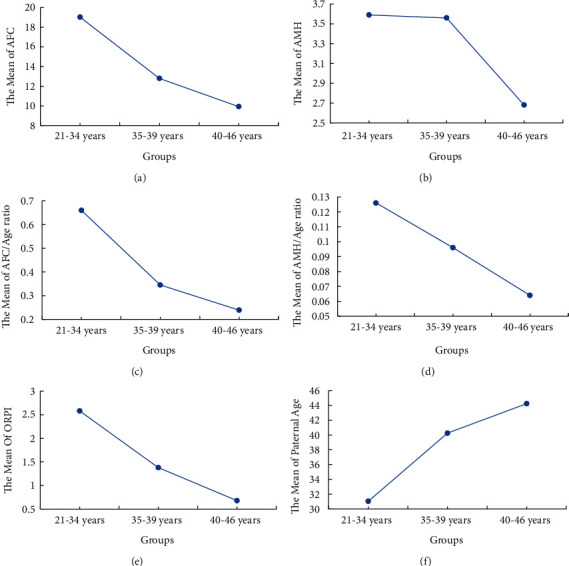
Mean plots of variables presenting the increasing/decreasing pattern of median values with respect to increasing age of subjects. (a) The decreasing pattern of AFC; (b) the decreasing pattern of AMH; (c) the decreasing pattern of AFC/age ratio; (d) the decreasing pattern of AMH/age ratio; (e) the decreasing pattern of ORPI; (f) the increasing pattern of paternal age. AFC, antral follicle count; AMH, anti-Müllerian hormone; ORPI, ovarian response prediction index.

Parameters such as the female age (years), duration of infertility (years), BMI, AFC, AMH levels, AFC/age ratio, AMH/age ratio, ORPI, NOR, GQE, paternal age, sperm concentration, and rate of normal morphological sperm among the three groups are given in Table 1. Multiple comparisons show that AFC, AMH levels, AFC/age ratio, AMH/age ratio, and ORPI decreased gradually with increase in age, while paternal age increased with increase in age (Figure 1). AMH decreased significantly with age between groups 1 and 3 (*P* < 0.01) and groups 2 and 3 (*P* < 0.001), as shown in Figure 1(b).

### 3.2. Pearson Correlation Analysis of All Parameters in Relation to NOR and GQE

NOR and GQE had significant statistical differences with AFC, AMH, AFC/age ratio, AMH/age ratio, and ORPI (*P* < 0.001), while NOR was positively correlated with LH (*P* < 0.05). However, NOR and GQE were negatively correlated with maternal age (*P* < 0.001 and *P* < 0.001, respectively) and the rate of good-quality embryos (*P* < 0.01 and *P* < 0.001, respectively), while NOR was negatively correlated with FSH (*P* < 0.001) (Table 2). All these parameters correlated with NOR could reflect the status of ovarian reserve and ovarian response.

### 3.3. ROC Curve Analysis for Forecasting the Numbers of Retrieved Oocytes

**Figure 2:**
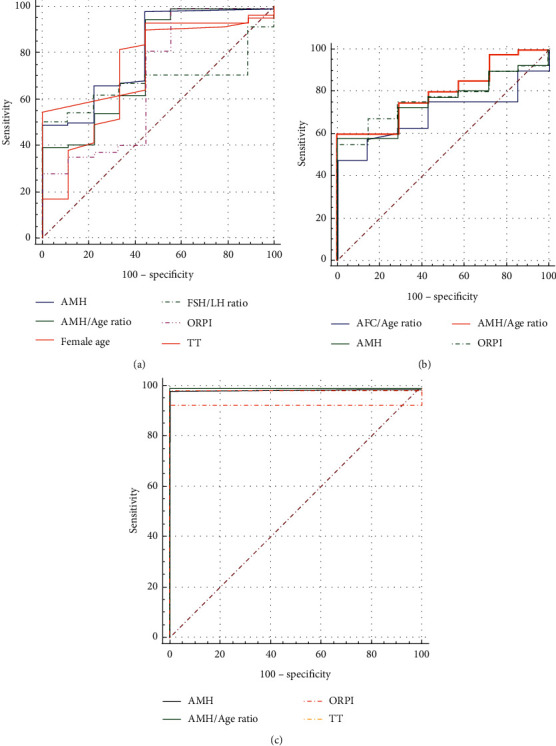
The comparison of variables evaluated oocyte retrieval on ROC curves. (a) ROC curves of variables evaluated oocyte retrieval in subjects aged 21–46 years. (b) ROC curves of variables evaluated oocyte retrieval in subjects aged 21–34 years. (c) ROC curves of variables evaluated oocyte retrieval in subjects aged 35–46 years. ROC curve, receiver operating characteristic curve; AFC, antral follicle count; AMH, anti-Müllerian hormone; FSH, follicle-stimulating hormone; LH, luteinizing hormone; ORPI, ovarian response prediction index; TT, total testosterone.

In one definition of poor ovarian response, the number of oocytes collected after conventional stimulation protocols was less than 4 [19, 28]. ROC curves were constructed to examine the performance of the variables in predicting the retrieval of >5 oocytes. An optimized threshold was determined, and the discriminative performance of the variables was assessed by AUC. The variables predicting oocyte retrieval concurrently in groups 1 and 2 were AMH, AMH/age ratio, and ORPI. Especially in group 2, these three variables reached a “high” grade in the diagnostic accuracy because AUC was significant (Table 3 and Figure 2). These variables performed better than other variables in assessing number of oocytes retrieval and provided an excellent predictive value in predicting ovarian response.

### 3.4. ROC Curve Analysis for Predicting the Numbers of Good-Quality Embryos

ROC curve analysis identified that neither of these variables could predict numbers of good-quality embryos (GQE) significantly in any group.

## 4. Discussion

The present study revealed a gradual decrease of AFC, AFC/age ratio, AMH/age ratio, and ORPI with increase in age of female where decrease in AMH was significant between groups 1, 2, and 3. The decreasing trend of aforementioned four variables is consistent with previous studies [4, 20, 29] and could accurately reflect the diminished status of ovarian reserve of infertile women with ageing. However, the trend of traditional predictors such as FSH, LH, E2, and FSH/LH ratio was not developed in this study. These predictors can be interpreted as the limitation of their predictive power in ovarian reserve of advanced-age infertile women.

A strong positive correlation was found between NOR, GQE, and AFC, AMH, AFC/age ratio, AMH/age ratio, and ORPI, while a strong negative correlation was found between NOR, GQE, and maternal age, the rates of good-quality embryos. These correlations are consistent with those reported previously [3, 11, 12, 20, 23, 30, 31]. These results supported the forecasting of NOR and prediction of GQE theoretically; however, the predictive accuracy and statistical significance needed to be evaluated by the ROC curve. Our results of ROC curves identified that the significant variables predicting oocyte retrieval were AMH, AMH/age ratio, and ORPI concurrently in the groups of all subjects; however, the AFC/age ratio was only significant in group 1, and AFC showed no significance in any of the groups. Previous studies reported that serum AMH levels were considered as the excellent predictor of quantitative aspects of assisted reproductive technology (ART), which had higher predictive accuracy for ovarian response and oocyte yield after ovarian stimulation than age or basal levels of FSH, E2, and inhibin B in clinical practice [5, 11, 18, 29, 32].

Other studies have reported that AFC has shown sufficient weekly and interobserver reliability in measuring ovarian reserve. However, AFC may be overestimated due to its inclusion of atretic follicles and, therefore, has no prognostic value for NOR and GQE [18]. Other markers including LH, E2, and inhibin B were weaker than FSH, AMH, and AFC [33]. AMH levels seemed to reflect the ovarian response and showed strong correlation with the number of AFC, NOC, and GQE [34]. Therefore, AMH and AFC may predict NOR and GQE in IVF [23, 35]. This study indicated that the predictive value of AFC and AFC/age ratio was limited; however, AMH and AMH related combined index had an excellent value for predicting NOR in reproductive-age women and advanced-age infertile women; this is potentially one of the conclusions of this study that has not been reported previously. Otherwise, other studies indicated that body mass index (BMI) and Gn dose affect the IVF success rates [36, 37]. However, we did not reach similar conclusions, which may be the reason for relatively few people within this study.

Nonsignificant results were observed in ROC curve for FSH, AMH, AFC, and related combined index predicting GQE in this study. Other factors, including sperm quality, oocyte quality, the status of oocyte fertilization and cleavage, culture medium type, and culture environment, affected the embryo development in vitro; therefore, GQE could not be predicted accurately simply via laboratory variables of infertile couples.

## 5. Conclusions

A decreasing trend of AFC, AMH, AFC/age ratio, AMH/age ratio, and ORPI with increase in age of female and a positive correlation of these parameters with NOR or GQE suggest these indexes are of great value in predicting ovarian function. Regarding oocyte retrieval, the predictive value of AFC and AFC/age ratio was limited; however, AMH, AMH/age ratio, and ORPI concurrently had an excellent value for predicting NOR in reproductive-age women and in advanced-age infertile women. Other variables including FSH, AMH, AFC, and related combined index predicting GQE were not significant statistically. This study demonstrated that AMH level and related combined index presented good predictive values for NOR and the suitability and individualization of conventional ovarian stimulation for in vitro fertilization.

## Figures and Tables

**Table 1:** Clinical data and variables' data of different age groups.

Variables	Age groups				*P* value^*∗*^
	Total subjects (*n* = 521), mean ± SD	Group 1 (21–34 years) (*n* = 64), mean ± SD	Group 2 (35–39 years) (*n* = 296), mean ± SD	Group 3 (40–46 years) (*n* = 161), mean ± SD	
Female age (years)	37.72 ± 4.25	29.11 ± 3.25	37.23 ± 1.15	42.06 ± 1.79	0.001
Duration of infertility (years)^#^	4.99 ± 3.94	3.92 ± 2.34	4.86 ± 3.70	5.74 ± 4.77	0.008
BMI (kg/m^2^)	23.41 ± 4.16	22.80 ± 3.10	23.33 ± 4.14	23.82 ± 4.57	0.254
AFC^#^	12.65 ± 6.99	19.02 ± 7.34	12.79 ± 7.00	9.92 ± 4.94	0.001
AMH (ng/ml)^#^	3.295 ± 2.55	3.59 ± 2.30	3.56 ± 2.82	2.68 ± 1.98	0.001
AFC/age ratio^#^	0.350 ± 0.221	0.660 ± 0.262	0.345 ± 0.192	0.239 ± 0.122	0.001
AMH/age ratio^#^	0.090 ± 0.072	0.126 ± 0.085	0.096 ± 0.076	0.064 ± 0.047	0.001
ORPI^#^	1.31 ± 1.51	2.58 ± 2.06	1.38 ± 1.52	0.68 ± 0.67	0.001
FSH (IU/liter)	7.08 ± 3.90	6.64 ± 3.88	7.12 ± 4.25	7.20 ± 3.16	0.636
LH (IU/liter)	4.52 ± 5.06	4.12 ± 2.92	5.02 ± 6.30	3.81 ± 2.64	0.051
FSH/LH ratio	2.28 ± 3.43	2.54 ± 2.74	2.19 ± 4.25	2.31 ± 1.30	0.781
E2 (pg/ml)	122.75 ± 349.98	90.17 ± 112.27	105.63 ± 287.77	168.49 ± 491.27	0.165
TT (nmol/liter)	1.06 ± 6.74	0.43 ± 0.19	1.55 ± 8.88	0.40 ± 0.19	0.453
NOR^#^	11.72 ± 6.77	12.03 ± 7.05	12.44 ± 7.25	10.27 ± 5.40	0.004
Fertilization rate (%)	76.18 ± 28.75	71.34 ± 21.26	79.28 ± 34.36	74.10 ± 20.73	0.130
GQE^#^	4.02 ± 3.09	4.18 ± 2.48	4.38 ± 3.46	3.31 ± 2.41	0.002
Rate of GQE (%)	56.78 ± 27.49	56.99 ± 23.66	57.08 ± 29.04	56.09 ± 27.38	0.964
Paternal age (years)^#^	40.35 ± 6.12	31.05 ± 4.13	40.25 ± 4.49	44.24 ± 5.29	0.001
Sperm concentration (10^6^/ml)^#^	55.45 ± 38.67	43.80 ± 51.14	59.57 ± 36.16	52.43 ± 36.26	0.006
PR (%)	46.08 ± 18.73	46.11 ± 22.41	46.01 ± 17.93	46.21 ± 18.70	0.994
Rate of normal morphological sperm (%)^#^	4.52 ± 1.29	3.59 ± 1.70	4.70 ± 1.09	4.57 ± 1.28	0.001
SDFI (%)	23.48 ± 15.57	22.30 ± 12.48	23.84 ± 17.12	23.39 ± 13.75	0.780

BMI, body mass index; AFC, antral follicle count; AMH, anti-Müllerian hormone; ORPI, ovarian response prediction index; FSH, follicle-stimulating hormone; LH, luteinizing hormone; E2, estradiol; TT, total testosterone; NOR, numbers of oocytes retrieved; GQE, numbers of good-quality embryos; SDFI, sperm DNA fragmentation index; PR, progressive motility (sperm); SD, standard deviation. ^*∗*^*P* value for the comparison between the three age subgroups. ^#^Multiple comparisons between the different two subgroups. Duration of infertility: group 1 vs. group 3 (*P* < 0.003); AFC, AMH/age ratio, AFC/age ratio, ORPI, and paternal age: all *P* < 0.001; AMH: group 1 vs. group 3 (*P* < 0.05), group 2 vs. group 3 (*P* < 0.001); NRO and GQE: group 2 vs. group 3 (*P* < 0.001, *P* < 0.001, respectively); sperm concentration: group 1 vs. group 2 (*P* < 0.003); normal morphology: group 1 vs. group 2 and group 1 vs. group 3 (all *P* < 0.001).

**Table 2:** Pearson correlation analysis of all variables in relation to NOR and GQE.

Variables	Pearson correlation	Related variables									
		Maternal age	AFC	AMH	AFC/age ratio	AMH/ age ratio	ORPI	FSH	LH	GQE	Rate of good-quality embryos
NOR	Coefficient	−0.168	0.501	0.428	0.465	0.442	0.573	−0.145	0.104	0.500	−0.149
	*P* value	0.001	0.001	0.001	0.001	0.001	0.001	0.002	0.024	0.001	0.010
GQE	Coefficient	−0.135	0.325	0.221	0.294	0.228	0.306	−0.071	0.031	NA	0.470
	*P* value	0.002	0.001	0.001	0.001	0.001	0.001	0.136	0.503	NA	0.001

AFC, antral follicle count; AMH, anti-Müllerian hormone; ORPI, ovarian response prediction index; FSH, follicle-stimulating hormone; LH, luteinizing hormone; NOR, numbers of retrieved oocytes; GQE, numbers of good-quality embryos.

**Table 3:** The ROC curve, AUC, and cutoff values of variables on predicting oocyte retrieval.

Subjects	Variables	AUC		Cutoff value	Sensitivity (%)	Specificity (%)
		Value (95% confidence interval)	*P* value			
All subjects^*∗*^	AMH	0.802 (0.738, 0.857)	＜0.0011	＞1.4	78.46	82.76
	AMH/age ratio	0.760 (0.692, 0.819)	＜0.0011	0.0279	85.77	72.41
	Maternal age	0.773 (0.706, 0.831)	0.0115	＞38	41.87	82.76
	FSH/LH ratio	0.670 (0.597, 0.737)	0.0460	≤1.331	32.63	100.00
	ORPI	0.684 (0.612, 0.750)	0.0011	＞0.261	82.03	63.16
	TT	0.728 (0.658, 0.790)	0.0323	＞0.16	91.20	55.56
21–34 years^#^	AFC/age ratio	0.691 (0.539, 0.818)	0.0171	＞0.741	47.50	100.00
	AMH	0.779 (0.634, 0.887)	＜0.0011	＞3.06	56.36	100.00
	AMH/age ratio	0.796 (0.654 0.900)	＜0.0011	＞0.096	58.18	100.00
	ORPI	0.768 (0.622, 0.878)	0.0012	＞1.927	55.00	100.00
35–46 years^†^	AMH	0.982 (0.948, 0.996)	＜0.0011	＞1.37	77.35	100.00
	AMH/age ratio	0.988 (0.957, 0.998)	＜0.0011	＞0.028	84.44	95.00
	ORPI	0.988 (0.957, 0.998)	＜0.0011	＞0.261	80.23	100.00
	TT	0.920 (0.868, 0.956)	＜0.0011	0.16	90.10	100.00

AFC, antral follicle count; AMH, anti-Müllerian hormone; AUC, the area under the curve; FSH, follicle-stimulating hormone; LH, luteinizing hormone; ORPI, ovarian response prediction index; ROC curve, receiver operating characteristic curve; TT, total testosterone. ^*∗*^Pairwise comparison of ROC curves: AMH vs. AMH/age ratio: *P* < 0.05; AMH vs. ORPI: *P* < 0.01; AMH/age ratio vs. ORPI: *P* < 0.05. ^#^Pairwise comparison of ROC curves: all *P*＞0.05. ^†^Pairwise comparison of ROC curves: AMH vs. TT: *P* < 0.05; AMH/age ratio vs. TT: *P* < 0.05; ORPI vs. TT: *P* < 0.05.

## Data Availability

The datasets that were used or analyzed during the current study are available from the corresponding authors on reasonable request.
